# Real life experience on the effect of Belimumab in patients with active systemic lupus

**DOI:** 10.1186/2193-1801-3-758

**Published:** 2014-12-22

**Authors:** Morton Scheinberg, Ricardo Golmia

**Affiliations:** Clinical Research Center Hospital AACD, São Paulo, 04032-060 Brazil

## Abstract

**Introduction:**

To evaluate real life experience on the effect of Belimumab in patients with active systemic lupus erythematosus.

**Case presentation:**

Twenty patinets with musclesketal symptoms were evaluated, three were discontinued for different resons and seventeen completed eight injections of Belimumab during a six month period. A fatigue score was also evaluated at the same time.

**Discussion and Evaluation:**

Marked reduction of disease activity and fatigue score were observed in the patient group.

**Conclusions:**

Belimumab is a novel form of therapy in patients with active systemic lupus erythematosus to be added in the standard of care particularly in patients with skin and joint symptoms.

## Introduction

Belimumab (trade name Benlysta) is a human monoclonal antibody that inhibits the cytokine B cell activating factor known as BAFF or BLYS and is to some extent responsible for the the increased autoantibody production observed in patients with systemic lupus erythematosus (SLE).(Zhang et al. 2001; Petri et al. 2008). It is approved for the treatment of SLE although clinical trials did not evaluate more severe disease such as severe lupus nephritis and central nervous manifestations (Furie et al. 2011).

In Brazil it was licensed for clinical use in July 2013. In this paper we report the safety and efficacy in 20 patients where Belimumab was indicated and completed six months of continuous use in a single center going from August 2013-July 2014.

## Material and Methods

Patients with active SLE according to the American College of Rheumatology (1997) were part of this study (Tan et al. 1982). They were on standard of care and with disease activity for more than sixty days attending the outpatient clinic. Disease activity was evaluated by the SELENA-SLEDAI(SS) score, and only patients with score above 8 had the indication to receive belimumab (Hawker et al. 1993). Anti DNA antibodies were measured by Elisa, complement leves by nephelometry.

Corticosteroid daily dose were recorded before during the study and after six months.

They received a total of eight injections of Benlysta intravenously in a six month period (10 mg per body weight). They were started on the period going from August 2013 till February 2014 and on July 2014 the six month period was completed. The functional assessment of chronic therapy fatigue (Facit fatigue 13 items) was employed before and after the the six month period.(Chandran et al. 2007). Questionnaires were applied to all 20 patients but only fifteen returned after the six month period of treatment with Benlysta. Statistical analyses were performed by the Wilcoxon paired t-test.

## Results

The demographics on the patient population are presented on Table 1. The drug was generally well tolerated, except for one patient that developed mild itching after the second injection and severe after the third injection. Three patients discontinue therapy for different reasons. One, for the loss of insurance coverage,one for the described side effect and the third for the presence of persistent disease activity (arthritis and fatigue). These symptoms were not not interpreted as flare but in fact lack of clinical response.

**Table 1:** **Demographics and disease characteristics in 20 patients with active SLE who were started on Belimumab**

Mean age	36+/- 9.2
Gender	Female 20
**Time diagnosis**	1- year 6pts
	1-5 years 11pts
	6-10 years 3 pts
**Clinical findings**	9 Pts joint and constitutional symptoms
	7 Pts skin and constitutional symptoms
	3 Pts joint and hematologic symptoms
**Ethnicity**	Caucasian 20pts

The mean age was 36 years and all patients were of Caucasian origin. Disease duration was variable and are depicted on the table.

The disease activity was composed of joint disease (9/17) cutaneous (7/17) hematologic (3/17) some patients had combination of joint and skin and only 15 had fatigue questionnaires before and after the six month period.

The results of the various parameters are depicted on Table 2. The mean SS score (10.2 + -1.1) reduced to (1.1 + - 1.2) anti ds DNA antibody from 180 to 60U, only 11 patients had anti DNA antibodies above the normal range (40U), C3 levels increased from 62 mg to 98 mg and corticosteroid dosage reduced from 20 mg to 7.5 mg/day.

**Table 2:** **Serology and steroid dose before and six months after eight injections of Belimumab**

Baseline	Six Months p Value
**Anti DNA (Mean +/- SD)** 180U + /-60	60 +/- 45U <0,05 (nl range inf. 40U/ml)
**C3 (Mean SD)** 63 mg +/-17	98 +/- 12U <0, 05 (nl range 90-153 mg/ml)
**Corticosteroid dose (Mean+/ - SD)** 20 mg+/-7.5 mg	7,5 mg+/-2.5 mg p <0,01

**Figure 1:**
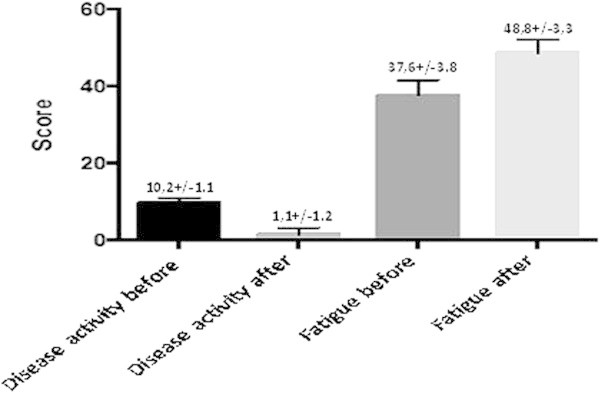
**Skin vasculitis before and after treatment.**

**Figure 2:**
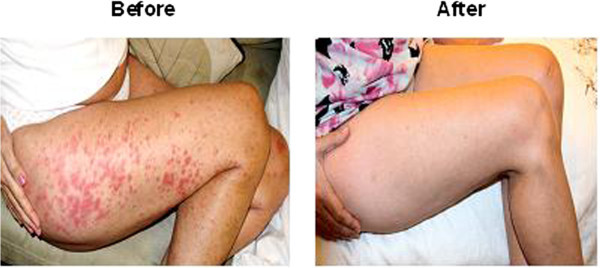
**Disease activity and fatigue scale before and after six months.**

The mean FACIT score was 37.6 + -3.8 and improved to 48.8 + -3.3 after six months of belimumab treatment.On these questionnaire score of 52 represents zero fatigue. In one of the patients with active joint and severe vasculitis only controlled with daily steroid dose ranging from 20 to 30 mg went into remission with monthly benlysta and 5.0 mg of daily steroid (Figure 1 (see Figure 2)).

## Discussion

In the present report we evaluate the efficacy of belimumab in a group of SLE patients attending the outpatient of a single Center and had completed six months treatment. Seventeen patients that were treated with belimumab showed marked reduction of SS, reduction in steroid daily use and in the majority of patients improvement in serology by reduction in anti DNA titers and increase in complement levels. Fatigue is an important symptom in patients with chronic diseases and SLE is no exception.

A number of self reported scales are used to measure fatigue, we used the FACIT scale composed of 13 items in this study.

Total score ranges from 0 to 52 and high scores represent less fatigue.

A significant improvement on fatigue scores were also observed in the group of patients that completed six months of treatment.

To our knowledge this is the first report in a full paper describing the short term efficacy and safety data in patients with SLE receiving belimumab and standard therapy in real life from a individual center. It was possible to observe that our results are similar to those described in clinical trials with long term evaluations, such as the seven years recently reported recently reported by Ginzler and co workers where it was possible to observe a sustained reduction of disease activity , improvement in serology and daily reduction on steroid use (Ginzler et al. 2014). In this initial report we were also able to show reductions in fatigue that correlated with scores of disease activity.

Fatigue improvement has not been reported in previous reports with Belimumab.

We conclude that belimumab therapy is associated with short treatment improvement in the majority of patients with lupus activity predominantly in muscleskeletal and mucocutaneous domains as recently suggested by Parodis and co workers (Parodis et al. 2013). Future management of lupus patients will include. Inhibitors of the BAFF-APRIL pathway, Belimubab being the first already in the market but it is expected that others under evaluation will be included in the future management of such patients with active disease and receiving steroids above the desirable dose (Isenberg et al. 2014; Furie et al. 2014).

## Conclusions

This is the first real life short term experience with a novel biologic therapy for the treatment of patients with active systemic lupus. The data here reported outlines the new perspectives of the introduction of Belimumab on the standard of care of patients with active disease.

### Patient declaration

All repeat All patients signed consent form to be part of this report.
